# Identification of Quinone Degradation as a Triggering Event for Intense Pulsed Light-Elicited Metabolic Changes in *Escherichia coli* by Metabolomic Fingerprinting

**DOI:** 10.3390/metabo11020102

**Published:** 2021-02-10

**Authors:** Qingqing Mao, Juer Liu, Justin R. Wiertzema, Dongjie Chen, Paul Chen, David J. Baumler, Roger Ruan, Chi Chen

**Affiliations:** 1 Department of Food Science and Nutrition, University of Minnesota, 1334 Eckles Ave, Saint Paul, MN 55108, USA; maoxx113@umn.edu (Q.M.); liux3514@umn.com (J.L.); wiert006@umn.edu (J.R.W.); chen5166@umn.edu (D.C.); dbaumler@umn.edu (D.J.B.); 2 Department of Bioproducts and Biosystems Engineering, University of Minnesota, 1390 Eckles Ave., Saint Paul, MN 55108, USA; chenx088@umn.edu (P.C.); ruanx001@umn.edu (R.R.)

**Keywords:** intense pulsed light, *Escherichia coli*, bacterial metabolome, membrane quinones, oxidative stress, intermediary metabolism

## Abstract

Intense pulsed light (IPL) is becoming a new technical platform for disinfecting food against pathogenic bacteria. Metabolic changes are deemed to occur in bacteria as either the causes or the consequences of IPL-elicited bactericidal and bacteriostatic effects. However, little is known about the influences of IPL on bacterial metabolome. In this study, the IPL treatment was applied to *E. coli* K-12 for 0–20 s, leading to time- and dose-dependent reductions in colony-forming units (CFU) and morphological changes. Both membrane lipids and cytoplasmic metabolites of the control and IPL-treated *E. coli* were examined by the liquid chromatography-mass spectrometry (LC-MS)-based metabolomic fingerprinting. The results from multivariate modeling and marker identification indicate that the metabolites in electron transport chain (ETC), redox response, glycolysis, amino acid, and nucleotide metabolism were selectively affected by the IPL treatments. The time courses and scales of these metabolic changes, together with the biochemical connections among them, revealed a cascade of events that might be initiated by the degradation of quinone electron carriers and then followed by oxidative stress, disruption of intermediary metabolism, nucleotide degradation, and morphological changes. Therefore, the degradations of membrane quinones, especially the rapid depletion of menaquinone-8 (MK-8), can be considered as a triggering event in the IPL-elicited metabolic changes in *E. coli*.

## 1. Introduction

*Escherichia coli* is a Gram-negative and facultative anaerobic bacterium widely distributed in the intestinal tracts of animals and humans [1,2]. Most *E. coli* strains are non-pathogenic, but Shiga-toxin-producing *E. coli* (STEC) strains, such as O157:H7 and O104:H4, have caused numerous cases of foodborne illnesses globally by contaminating vegetable, fruit, beverages, and meat [3]. Therefore, effective disinfection of pathogenic *E. coli* is highly desirable in human food and animal feed productions.

Thermal pasteurization, which raises the internal temperature of food to at least 70 °C, is the most commonly used technology to disinfect *E. coli* in food manufacturing [4]. Despite its effectiveness, thermal pasteurization is known to alter the physical and chemical properties of food [5]. Other alternative sterilization technologies, including pulsed magnetic field [6], capillary discharge plasma [7], high hydrostatic pressure [8], high pressure carbon dioxide [9], and ultraviolet-C (UV-C) irradiation [10], have been developed for food manufacturing, but their applications in disinfecting the dry food with a low water activity (a_w_), are restricted by their low efficiency and poor compatibility with industry practices [11].

Intense pulsed light (IPL) is a novel non-thermal disinfection technology containing a broad spectrum of ultraviolet (UV), visible, and infrared light. A prototype IPL platform for the inactivation of microbes in low a_w_ food was developed recently and its efficacy has been shown in disinfecting non-fat dry milk powder [11] and mesquite pod flour [12]. The IPL system inactivates microbes by delivering intensive energy to the target surface through pulsed flashing (1–20 flashes per second) of broad-spectrum light (190–1100 nm) [13]. The mechanisms of IPL-elicited bactericidal and bacteriostatic effects have been examined previously. UV irradiation, making up 25% of the IPL wavelength spectrum, contributes to its pasteurization effect via photochemical changes [14] and DNA lesions [15]. In addition, IPL disrupts cell structural and membrane integrity, leading to the leakage of intracellular content and ultimately cellular death [16].

Current understandings on the bactericidal and bacteriostatic effects of IPL were mainly derived from targeted analyses of DNA, proteins, and cellular structures through gel electrophoresis [16], scanning electron microscopy [14], confocal laser scanning microscopy, and flow cytometry [15]. Despite the importance of metabolism in cell proliferation and survival, especially under stresses and challenges, little information on IPL-elicited metabolic changes in bacteria has been revealed by these studies. Metabolomics, as a highly effective analytical platform, can meet this need for examining the global changes in microbial metabolism under challenges [17]. Metabolomic analysis has been applied to study the mechanisms of bactericidal treatments, including electrolyzed water and mild heat [18], halogenated compounds [19], and titanium dioxide nanoparticles [20]. In this study, IPL-elicited metabolic changes in *E. coli* K-12, a representative *E. coli* strain, were examined by liquid chromatography–mass spectrometry (LC-MS)-based metabolomics. The IPL-responsive metabolites were identified, and the associated metabolic events were characterized.

## 2. Results

### 2.1. Effects of IPL on E. coli Growth and Morphology

**Figure 1:**
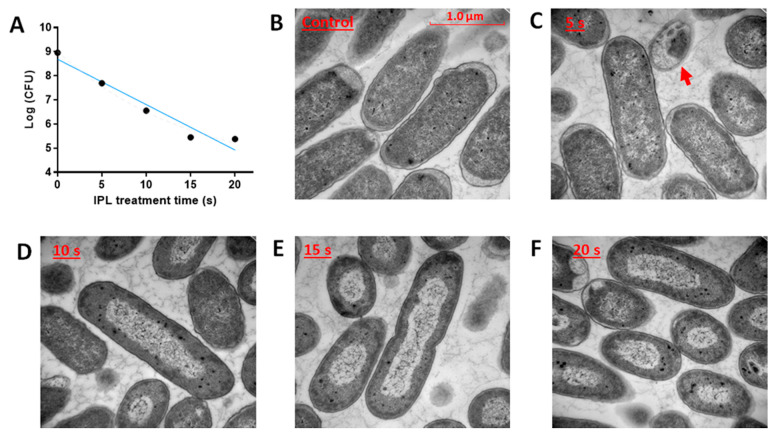
Effects of IPL on *E. coli* growth and morphology. (**A**) Plate counts of *E. coli* after the IPL treatments. The linear regression between Log-transformed CFU and the IPL treatment time (*t*) yielded an equation of Log(CFU) = −0.1882 *t* + 8.688 with r^2^ = 0.94. The control and IPL-treated *E. coli* cells were examined by TEM imaging: (**B**) control; (**C**) 5 s (a dead cell is marked by the red arrow); (**D**) 10 s; (**E**) 15 s; and (**F**) 20 s. Scale bars = 1.0 μm.

The influence of IPL treatments on *E. coli* growth was evaluated by measuring the colony-forming units (CFU). Dose-dependent decrease of CFU was observed and about four log reduction of CFU was achieved with 15 s of IPL treatment (Figure 1A). The morphology of IPL-treated *E. coli* cells was evaluated by the transmission electron microscopy (TEM) analysis. Compared to relatively uniform control cells (Figure 1B), IPL-treated *E. coli* cells varied in size and shape due to visible disruptions in cellular structures, mainly in nucleoid and cell walls (Figure 1C–F). At 5 s, the disruption of the cellular inner structure was evidenced by partially blurry and rough cell walls and the appearance of a dead cell body with reduced cytoplasmic content (Figure 1C). At 10, 15, and 20 s, these IPL-elicited morphological changes were intensified, altering *E. coli* cells from rod-shaped to spherical-shaped with a thickening cell wall (Figure 1D–F). More importantly, the electron density in the nucleoid region was reduced time-dependently and the continuous DNA strands became apparent, indicating the separation of genetic contents from other cytoplasmic contents and potential changes in chromosome structure (Figure 1C–F).

### 2.2. Modeling and Identification of IPL-Induced Changes in E. coli Metabolome

**Figure 2:**
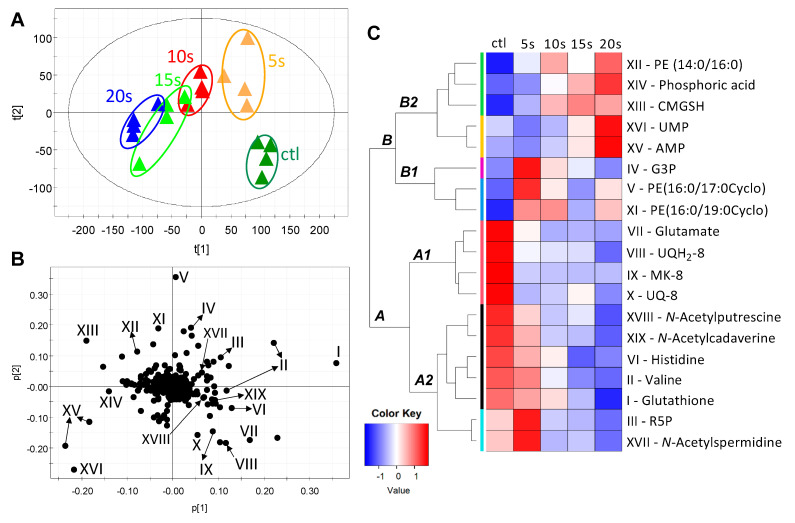
Modeling of IPL-elicited changes in the *E. coli* metabolome. Pooled data from the LC-MS analysis of organic and aqueous extracts of *E. coli* cells were processed by principal components analysis (PCA). (**A**) Scores plot of the PCA model. The samples in the same treatment group (*n* = 4) are circled. A scores plot containing three quality control (QC) samples is presented in Figure S2. (**B**) Loadings plot of the PCA model. The labeled markers (I–XIX) are the identified metabolites that contribute to the separation of sample groups. Their identities are listed in Table 1. (**C**) Heatmap from the clustering analysis of IPL-responsive markers (I–XIX). PE, phosphatidylethanolamine; CMGSH, *S*-carboxymethyl-glutathione; UMP, uridine monophosphate; AMP, adenosine monophosphate; G3P, glycerol 3-phosphate; UQH_2_-8, ubiquinol-8; MK-8, menaquinone-8; UQ-8, ubiquinone-8; R5P, ribose 5-phosphate.

The metabolic status of *E. coli* cells was examined by the LC-MS-based metabolomic analysis of both lipids and hydrophilic metabolites in the respective organic and aqueous extracts. The distribution of control and IPL-treated samples in the principal components analysis (PCA) model on the detected metabolites indicated the progressive changes in the *E. coli* metabolome after the IPL treatments (Figure 2A and Figure S2). The metabolites contributing to this time-dependent separation were identified in a loadings plot (Figure 2B), and their structural identities were defined by elemental composition analysis, database search, MSMS fragmentation, and the comparisons with authentic standards (Table 1). The correlations among these metabolite markers were further characterized by a hierarchical cluster analysis (HCA), producing a heatmap based on their abundances across all treatment groups (Figure 2C). Two major clusters of IPL-responsive metabolites (labeled as A and B in Figure 2C) were observed in the heatmap, in which Cluster A contains the metabolites decreased by IPL while Cluster B mainly contains the metabolites increased by IPL in at least one IPL treatment. These IPL-responsive metabolites were further characterized by their relative abundances in *E. coli* cells as well as their structures and metabolic functions.

### 2.3. Influences of IPL Treatment on Membrane Lipids

**Figure 3:**
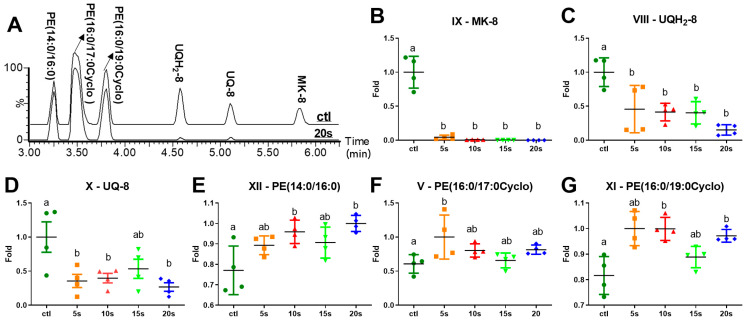
Influences of IPL treatment on membrane lipids. (**A**) Representative chromatographs on membrane phospholipids and quinones in the organic fractions of control and IPL-treated *E. coli* (20 s). The relative abundances of lipid markers across all the samples were compared by arbitrarily setting the average of the treatment group with the highest relative abundances as 1: (**B**) menaquinone-8 (IX); (**C**) ubiquinol-8 (VIII); (**D**) ubiquinone-8 (X); (**E**) PE(14:0/16:0) (XII); (**F**) PE(16:0/17:0Cyclo) (V); and (**G**) PE(16:0/19:0Cyclo) (XI). Different letters (a and b) indicate significant differences (*p* < 0.05) between timepoints.

As shown in the representative LC-MS chromatograms of extracted *E. coli* lipids, IPL dramatically decreased menaquinone-8 (MK-8; IX), ubiquinol-8 (UQH_2_-8; VIII), and ubiquinone-8 (UQ-8; X), which are three membrane-bound isoprenoid quinones and essential electron carriers in the respiratory chain (Figure 3A). This observation is consistent with the PCA modeling and clustering analysis, in which MK-8, UQH_2_-8, and UQ-8 form a subcluster of metabolites (A1) in the heatmap that were decreased by the IPL (Figure 2C). Among them, MK-8 was almost diminished at 5 s of IPL treatment and became undetectable afterwards (Figure 3B). The levels of UQH_2_-8 and UQ-8 were decreased dramatically at 5 s and then stabilized afterwards (Figure 3C,D). In contrast, the relative abundances of PE(14:0/16:0) (XII), PE(16:0/17:0Cyclo) (V), and PE(16:0/19:0Cyclo) (XI), three dominant phospholipid species in the *E. coli* membrane (Figure 3E–G), were either unchanged or increased in the IPL treatments, suggesting that the IPL-elicited changes in membrane quinones were selective and specific.

### 2.4. Influences of IPL Treatment on Cytoplasmic Metabolites

**Figure 4:**
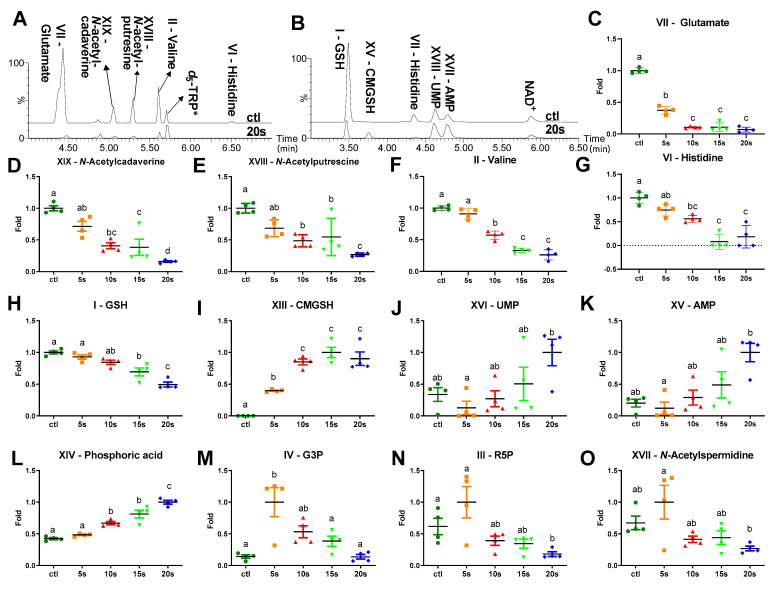
Influences of IPL treatment on cytoplasmic metabolites. (**A**) Representative chromatographs on amino acid metabolites in the aqueous fractions of control and IPL-treated *E. coli* (20 s). These metabolites were detected via dansyl chloride derivatization. *d_5_*-TRP (*d_5_*-tryptophan) was the internal standard in amino acid analysis and had stable signals across all the samples. (**B**) Representative chromatographs on hydrophilic metabolites in the aqueous fractions of control and IPL-treated *E. coli* (20 s). These metabolites were detected in the negative ionization mode without derivatization. The relative abundances of cytoplasmic metabolite markers across all the samples were compared by arbitrarily setting the average of the treatment group with the highest relative abundances as 1. The peak of NAD^+^ is included in the chromatograph for its relative stable signals across all the samples: (**C**) glutamate; (**D**) *N*-acetylcadaverine; (**E**) *N*-acetylputrescine; (**F**) valine; (**G**) histidine; (**H**) GSH, glutathione; (**I**) CMGSH, S-carboxymethyl-glutathione; (**J**) UMP, uridine monophosphate; (**K**) AMP, adenosine monophosphate; (**L**) phosphoric acid; (**M**) G3P, glycerol 3-phosphate; (**N**) R5P, ribose 5-phosphate; and (**O**) *N*-acetylspermidine. Different letters (a–d) indicate significant differences (*p* < 0.05) between timepoints.

The LC-MS analysis of hydrophilic metabolites in the cytoplasm of *E. coli* was conducted through both hydrophilic-interaction chromatography (HILIC) and dansylation derivatization for amino acids. As shown in the representative chromatograms of aqueous *E. coli* extracts, IPL elicited diverse metabolic changes in the cytoplasm (Figure 4A,B). These cytoplasmic metabolites could be divided into three groups based on the patterns of IPL-elicited changes because some of them were either consistently decreased or increased by IPL, while others were increased first and then decreased afterwards (Figure 2C and Figure 4C–O).

Among the detected amino acids and their derivatives, many of them underwent time- and dose-dependent decreases after the IPL treatments. The change in glutamate was the most dramatic among them, as its level was significantly lowered at 5 s and almost diminished at 10 s (Figure 4C). The progressive decreases of valine, histidine, and acetylated polyamines, including *N*-acetylputrescine and *N*-acetylcadaverine, were also observed after 10 s of the IPL treatment (Figure 4D–G). Furthermore, glutathione (GSH), a tripeptide antioxidant, was also decreased, but at a slower pace than the amino acids and polyamines, as the significant decreases of GSH did not occur until 15 s of IPL (Figure 4H).

In contrast to the decreases in amino acid metabolites and GSH, S-carboxymethyl-glutathione (CMGSH), a GSH metabolite, was undetectable before the IPL treatment, but dramatically increased afterwards (Figure 4I). Its identity was confirmed by the synthesized standard (Figure S1). In addition, nuclear acid metabolites, including adenosine monophosphate (AMP), uridine monophosphate (UMP), and phosphoric acid, were gradually increased after the IPL treatments (Figure 4J–L).

Interestingly, glycerol 3-phosphate (G3P), ribose 5-phosphate (R5P), and *N*-acetylspermidine displayed a similar pattern as they were increased at 5 s and then decreased by longer IPL treatments (Figure 4M–O). Among them, the most dramatic change occurred to G3P since its level was elevated by about five folds after 5 s of IPL (Figure 4M).

## 3. Discussion

**Figure 5:**
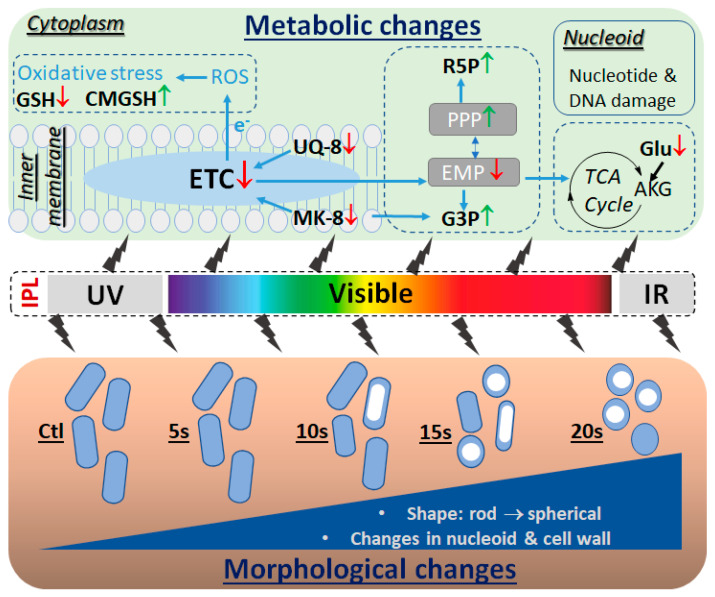
Summary of IPL-elicited morphological and metabolic changes in *E. coli*. IPL covers ultraviolet (UV), visible, and infrared (IR) light. IPL-treated *E. coli* cells underwent changes in size, shape, and structures, especially in the nucleoid. Metabolic changes occurred in the inner plasma membrane, cytoplasm, and nucleoid of *E. coli* after the IPL treatments, affecting the metabolites in ETC, oxidative stress, Embden–Meyerhof–Parnas glycolytic pathway (EMP), pentose phosphate pathway (PPP), amino acid, and nucleotide metabolism. Among them, the degradation of quinone electron carriers, especially the depletion of MK-8, is the earliest and most dramatic metabolic event induced by the IPL.

As a light-based irradiation disinfection method, IPL enables the disruption of different chemical bonds in target molecules by the photoenergy from its broad spectrum of wavelengths. Therefore, it is not surprising that diverse metabolic changes in the *E. coli* metabolome were observed after the IPL treatments in this study. Some metabolic changes are the direct consequences of photochemical reactions on the metabolites with light-sensitive chemical bonds while others are the consequential effects on their downstream biochemical pathways. Analyzing the time course and scale of these metabolic changes as well as the biochemical connections among them revealed a cascade of events that might be initiated by the degradation of quinone electron carriers and then followed by the oxidative stress, disruption of intermediary metabolism, nucleotide degradation, and morphological changes. Potential causes and functions of these metabolic events are summarized (Figure 5) and discussed as follows.

### 3.1. Degradation of Quinone Electron Carriers

MK-8 is a naphthoquinone in the vitamin K family while UQH_2_-8, and UQ-8 are a pair of reduced and oxidized benzoquinones in the coenzyme Q family [21]. With eight isoprene units in their hydrophobic tails, all three quinones are embedded in the cytoplasmic (inner) membrane of *E. coli* as the electron carriers in the electron transport chain (ETC) [22]. Dramatic decreases of MK-8, UQH_2_-8, and UQ-8 were among the earliest responses of *E. coli* to IPL. This effect was selective since PEs in the *E. coli* membrane, which typically contain C14:0, C16:0, C17:0Cyclo, and C19:0Cyclo as their main fatty acids [23,24], were not decreased by the treatment. The sensitivity of these isoprenoid quinones to photo-oxidation has been documented. MK-8 is highly sensitive to near-UV light (300–380 nm) because the dose of 334 nm light to deplete the MK-8 in *E. coli* was much lower than the dose for killing [25]. UQ-8 and UQH_2_-8 in *E. coli* also undergo degradation under near-UV light [26], but their resistance to photoenergy is greater than MK-8 [25]. The observation of more rapid and complete degradation of MK-8 than UQH_2_-8 and UQ-8 in the IPL-treated *E. coli* in this study is consistent with these observations (Figure 3B–D). As electron carriers, MK-8 and UQ-8 play essential roles in anaerobic and aerobic respiration, respectively [25,27]. MK-8 deficiency has been shown to delay the growth of *E. coli* [25]. Additionally, *E. coli* mutant depleted of MK-8 and UQ-8 could not grow in either anaerobic or aerobic conditions [28]. The IPL-elicited deleterious effect on MK-8 and UQ-8 is hence essential for the inactivation of *E. coli* and preventing further growth of the remaining viable cells (Figure 5).

### 3.2. Direct Stress Responses

As the reduced form of UQ-8, UQH_2_-8 is an antioxidant and a scavenger of reactive oxygen species (ROS) [25,27]. Therefore, the degradation of quinone electron carriers is expected to negatively affect the antioxidant capacity of *E. coli*. More importantly, electron leakage is a deemed consequence of quinone degradation and ETC dysfunction, leading to the propagative formation of ROS. Indeed, the occurrence of oxidative stress in the IPL-treated *E. coli* cells was indicated by the changes in glutathione (GSH) and CMGSH (Figure 5). GSH, as the most abundant antioxidants in *E. coli*, experienced gradual loss upon IPL treatment (Figure 4H). In contrast, CMGSH, a glutathione derivative absent in untreated *E. coli*, started to present and then accumulated progressively after the IPL treatment (Figure 4I). The formation of CMGSH is likely to be a spontaneous response to the IPL-elicited oxidative stress through a reaction between an electrophile and GSH. The reaction could be mediated by glutathione s-transferases (GSTs), which are ubiquitous in *E. coli* for catalyzing the conjugation of electrophiles with the thiol residue of GSH [29], but also non-enzymatic according to the mild reaction condition required in the standard synthesis (Section 4.8). The identities of its electrophilic precursors are unknown. A previous study has shown that CMGSH was synthesized in *E. coli* treated with iodoacetate [30]. Given their absence in the *E. coli* culture, iodine and iodoacetate are unlikely precursors. Since chloride and acetate are constitutive components in *E. coli*, whether the IPL-derived photocatalysis could facilitate the formation of CMGSH from chloroacetate or other electrophilic species requires further investigation. Nevertheless, the formation of CMGSH is a prominent metabolic feature of IPL treatment and could become a potential marker of oxidative stress in *E. coli*. Besides oxidative stress, the transient increase of *N*-acetylspermidine at 5 s of IPL treatment (Figure 4O) might indicate the thermal stress of IPL. Acetylation of spermidine in *E. coli* was found to be responsive to heat, cold shock, and high pH [31]. Considering the intense photoenergy of IPL, the occurrences of heat shock-related metabolic events are expected in the affected *E. coli* cells.

### 3.3. Disruption of Intermediary Metabolism

A functioning ETC is essential for normal intermediary metabolism. The Embden-Meyerhof–Parnas glycolytic pathway (EMP), the Entner–Doudoroff pathway (ED), the pentose phosphate pathway (PPP), and the tricarboxylic acid (TCA) cycle are the central intermediary metabolism pathways in *E. coli* [32]. The observed changes in G3P, R5P, glutamate, and other amino acids reflect the significant impacts of IPL on these pathways, potentially through the disruption of ETC (Figure 5). An immediate metabolic consequence of rapid MK-8 depletion in *E. coli* could be the simultaneous increase of G3P at 5 s of IPL. G3P is the substrate of glycerol-3-phosphate dehydrogenase, which functions as the primary dehydrogenase in the anaerobic respiration of *E. coli* by catalyzing the oxidation of glycerol-3-phosphate to dihydroxyacetone phosphate and passing the electron to membrane-bound MK-8 [33]. As MK-8 is diminished at 5 s in IPL-treated *E. coli*, the electron flow was expected to be significantly shunted, leading to the acute accumulation of G3P. The downregulation of glycolysis in IPL-treated *E. coli* cells may lead to the activation of the PP pathway, especially its non-oxidative phase that does not depend on electron transport. The observed increase of R5P potentially reflects this influence of IPL on the PP pathway since R5P is a terminal metabolite from the non-oxidative phase of PP and a precursor for nucleic acid synthesis. In fact, this diverted flux from glycolysis to PP pathway, marked by the increased production of R5P, has been characterized as the first response to UV-induced oxidative stress in eukaryotic cells [34] as well as a coping mechanism of *E. coli* in response to H_2_O_2_-induced oxidative stress [35].

After the IPL treatment, glutamate, together with *N*-acetylated polyamines and other amino acids, were decreased or depleted in *E. coli* (Figure 4C–G). Glutamate and polyamines are mainly produced from nitrogen assimilation, thereof functioning as nitrogen deposits in *E. coli* [36]. In fact, glutamate is the most abundant free amino acid in *E. coli* [37]. The studies on eukaryotic cells have shown that rapid decreases of intracellular amino acids, especially glutamate, occurred when the ETC was inhibited [38,39]. Glutamate catabolism was mainly catalyzed by aspartate transaminase (AST), which converts glutamate to α-ketoglutarate (AKG) via transamination with oxaloacetate, producing aspartate [40]. The relevance of this metabolic event to survival and proliferation was demonstrated by the fact that the cells deficient in AST died rapidly when ETC was inhibited [38]. Since AST is expressed in *E. coli*, it is likely that the glutamate flux in the IPL-treated *E. coli* was quickly diverted for the metabolic routes required for survival following the inhibition of ETC. It should be noted that the intracellular aspartate in the current study was below the detection limit. Therefore, whether the AST activity was responsible for the IPL-induced decrease of glutamate needs further study. Alternatively, glutamate might be catabolized by glutamate dehydrogenase (GDH), which reversibly converts glutamate to α-ketoglutarate and ammonia [41]. GDH was upregulated for AKG production in H_2_O_2_-challenged *Pseudomonas fluorescens*, a common Gram-negative bacterium [41,42]. AKG is an important branching point in cellular metabolism, connecting amino acid, energy production, carbohydrate, and lipid metabolic pathways [43]. More importantly, AKG can act as a ROS scavenger [42,44]. Moreover, histidine, which was decreased by the IPL (Figure 4G), can also move into the AKG pool as a precursor for glutamate [42]. Further investigations are needed to determine the exact metabolic fate of glutamate after the IPL treatments.

### 3.4. Nucleotide Degradation and Morphological Changes

The elevation of nucleotide degradation products, including AMP, UMP, and phosphoric acid, was observed in the IPL-treated *E. coli* (Figure 4J–L). Compared to other dramatic metabolic changes (Figure 5), the increases in AMP and UMP, two monophosphate metabolites from ATP and UTP, only occurred after the prolonged IPL exposure. These observations suggested the occurrence of mild DNA damage and nucleotide degradation, which are expected for irradiation treatments, including IPL [15], but not extensive degradation or destruction. This conclusion was supported by the observation of continuous DNA strands in the TEM imaging (Figure 1B–F) and a preliminary random amplified polymorphic DNA-PCR (RAPD-PCR) test on the genomic DNA of these IPL-treated *E. coli*, in which significant IPL-dependent DNA damage was not observed (data not shown).

Despite limited impacts on the integrity of genetic entities, the transcriptional activities of *E. coli* were likely disrupted by the prolonged IPL exposure due to the clear separation of nucleoid from other cytoplasmic contents (Figure 1D–F). This assumption is based on the known influences of the altered ribosome and nucleoid distribution on the translational and transcriptional activity in *E. coli* [45]. These effects, together with other morphological features, including the alterations in cell shape and relatively intact membrane (Figure 5), suggest that some of IPL-treated *E. coli* cells might enter the viable but nonculturable (VBNC) state, instead of cell death. Similar morphological features, including the presence of low electronic density area and smaller spherical shape, have been observed in the *E. coli* under the VBNC states induced by high pressure CO_2_ [46] and low temperature [47,48]. Previous studies on the biochemical features of VBNC state have shown that *E. coli* cells exposed to high-pressure CO_2_ had low metabolic and transcriptional activities and shared some similar metabolic changes observed in the current study, including decreased glycolysis and increased amino acid catabolism for energy supply as well as elevated AMP production [49]. Because the bacteria in the VBNC state may be resuscitated under appropriate conditions [50], further tests are needed to determine the status of IPL-treated *E. coli* cells as well as the impacts on bactericidal and bacteriostatic activities of this irradiation technology.

### 3.5. Potential Issues Related to the IPL Application in Food Disinfection

Pathogenic microbes in food could exist in different states (dormancy and growth) and phases (lag, exponential, stationary, and death phases in growth). The *E. coli* from the overnight culture in this study was more likely in the stationary phase. In general, *E. coli,* as well as other bacteria, in this phase is more resistant to stressors [51], such as UV exposure [52], and has higher survival rate than its counterpart in the exponential phase due to less metabolic activities [53] and greater DNA repair capacity [54]. Therefore, the IPL treatment might be more effective in disinfecting *E. coli* in the exponential growth phase. On the other hand, the main purpose of the IPL platform in this study is for disinfecting powdered food with low a_w_, in which bacteria exist mostly in the dormant state. In this state, many bacteria, including *E. coli*, have very low metabolic activity and higher resilience to challenges, such as antibiotics [55], hydrogen peroxide, and heat stress treatments [56]. The influences of IPL on the metabolome of dormant bacteria require further studies. Considering quinones and many IPL-sensitive metabolites are constitutive components of bacterial cells, negative impacts of IPL on the metabolism and cultivability of treated bacteria in powdered food are expected. In fact, our recent studies have shown that the IPL treatment effectively inactivated *Cronobacter sakazakii* in non-fat dry milk and *Bacillus cereus* spores in mesquite pod flour [11,12].

The successful adoption of the IPL platform in food processing and manufacturing will be largely dependent on its disinfection efficacy as well as its impacts on food quality. Chemometric analyses have been conducted to examine the effects of IPL on non-fat dry milk powder and mesquite pod flour [11,12]. The treatment did not significantly alter the color, amino acid composition, and the levels of volatile off-flavor compounds in milk powder [11]. Mesquite pod flour retained the color and had reduced levels of volatile off-flavor compounds after the IPL treatment, though minor lipid oxidation was observed [12]. Overall, IPL shows potential as an effective tool for disinfecting powdered foods.

To our knowledge, this is the first attempt at analyzing the cellular metabolism of IPL-treated *E. coli* via the metabolomics platform. The decreases in quinone electron carriers, especially the rapid depletion of MK-8, can be considered as the most prominent metabolic event due to its contribution to oxidative stress and its connections with intermediary metabolism. Further investigations on the mechanisms of IPL-elicited metabolic effects and their contributions to its efficacy will provide insights for the future development of the IPL platform with improved pasteurization capability.

## 4. Materials and Methods

### 4.1. Culture of E. coli

*E. coli* strain K-12 W3110 (ATCC 27325) was chosen as the surrogate of food-borne pathogens to examine the IPL-elicited bacteriostatic and bactericidal effects. After culturing the K-12 from a frozen stock on TSA agar medium, a single colony was chosen to inoculate Luria–Bertani broth (EMD Millipore, Billerica, MA, USA). After 12-h incubation at 37 °C in a rotary shaker set to 200 rpm, *E. coli* cells were harvested at an optical density (OD_600_) around 1, and then centrifuged at 7500× *g* for 10 min in 50 mL centrifuge tubes. After decanting the supernatant, the pellets were washed with phosphate buffered saline (PBS), and then re-suspended to the volume of bacterial culture for IPL treatments.

### 4.2. Chemicals

LC-MS-grade water and acetonitrile (ACN) were purchased from Fisher Scientific (Houston, TX, USA). Dansyl chloride (DC), *n*-butanol, acetone, *d_5_*-tryptophan, tripentadecanoin, sulfadimethoxine, and amino acid standards were purchased from Sigma-Aldrich (St. Louis, MO, USA).

### 4.3. IPL Treatment of E. coli

For each IPL treatment, a 30 mL K-12 suspension in PBS was transferred to a petri dish (15 cm diameter) and then placed under the light source (wavelength 190–1100 nm) of a Z-1000 SteriPulse-XL system (Xenon Corporation, Woburn, MA, USA). The frequency, pulse width, and fluence of IPL treatment were 3 pulse/s, 360 μs/pulse, and 1.27 J/cm^2^ per pulse at a distance of 8 cm from the quartz window, respectively. The 5-, 10-, 15-, and 20-s treatments correspond to 19.05, 38.1, 57.15, and 76.2 J/cm^2^ photoenergy, respectively. After the IPL treatment, the *E. coli* suspension was immediately chilled on ice and then transferred to a centrifuge tube for further analysis.

### 4.4. Counting of Surviving Cells

The numbers of cells existing in the control and treated samples were measured by using 3M Petrifilm™ Aerobic Count Plates (3M Health Care, St. Paul, MN, USA). These plates were proven to yield equivalent results to the standard pour plates for aerobic count plate enumeration [57]. Samples were serially diluted with PBS to obtain countable colonies and then plated in duplicate on the count plates.

### 4.5. Morphological Analysis

Transmission electron microscopy (TEM) was used to examine the structural and morphological changes of *E. coli*. After the IPL treatment, the pellet was obtained by the centrifugation at 5000× *g* at 4 °C for 10 min, and then washed with PBS twice prior to being fixed in 2% glutaraldehyde. Samples were then submitted to the University of Minnesota Imaging Center for the TEM analysis.

### 4.6. Sample Preparation for LC-MS Analysis

The control and IPL-treated *E. coli* samples were centrifuged at 5000× *g* at 4 °C for 10 min. The cell pellet was washed with PBS twice, and then re-suspended in a 0.5 mL of methanol solution, containing 50 µM *d_5_*-tryptophan, 10 µM tripentadecanoin, and 1 µM sulfadimethoxine as the internal standards for monitoring LC-MS performance. The methanol suspension was vortexed and sonicated for 30 s, and then mixed with 0.5 mL of chloroform and 0.4 mL of H_2_O. The mixture was centrifuged at 14,000× *g* at 4 °C for 10 min for phase separation. The upper polar fraction was transferred to a new tube and stored at −80 °C prior to further analysis. The bottom non-polar fraction was dried under nitrogen, reconstituted in 0.5 mL of *n*-butanol, and then stored at −80 °C prior to further analysis.

### 4.7. Chemical Derivatization

To detect amino acids, the polar fraction of *E. coli* extract was derivatized with dansyl chloride (DC) prior to LC-MS analysis. Briefly, 5 μL of *E. coli* polar fraction were mixed with 5 μL of water, 50 μL of 10 mM sodium carbonate, and 100 μL of DC (3 mg/mL in acetone). The mixtures were incubated at 60 °C for 15 min and centrifuged at 14,000× *g* for 10 min. The supernatant was transferred to a HPLC vial for LC-MS analysis.

### 4.8. Synthesis of S-carboxymethyl-glutathione (CMGSH)

CMGSH was synthesized according to a published method [58]. Briefly, 100 μL of 100 μM glutathione were mixed with 100 μL of 10 mM iodoacetic acid in 10 mM ammonium bicarbonate buffer (pH = 10, adjusted with ammonium hydroxide). After the incubation at room temperature for 1 h, the formation of CMGSH was confirmed by the LC-MS analysis of the reaction mixture.

### 4.9. LC-MS Analysis

The workflow of LC-MS analysis was described previously [59,60]. In brief, 5 μL of aliquot were injected into an Acquity ultra-performance liquid chromatography system (Waters, Milford, MA, USA) and then separated in a UPLC column in a 10-min run at flow rate of 0.5 mL/min. The polar fraction and its amino acid derivatives were separated by a BEH Amide column and a BEH C18 column, respectively. The non-polar fraction was separated by a BEH C8 column. The mobile phase gradient used for each column is listed in Table S2. The LC elute was directly introduced in a XEVO-G2-S QTOF mass spectrometer (Waters) for accurate mass measurement and ion counting. The conditions of electrospray ionization (ESI) detection are listed in Table S3. For accurate mass measurement, the mass spectrometer was calibrated with sodium formate (range *m*/*z* 50–1200) and monitored by intermittent injection of the lock mass leucine encephalin ([M + H]^+^ = *m*/*z* 556.2771, [M − H]^−^ = *m*/*z* 554.2615) for polar fraction or reserpine ([M + H]^+^ = *m*/*z* 609.2812) for non-polar fraction. Chromatograms were acquired and processed by MassLynx^TM^ (Waters, Milford, MA, USA). Tandem MS (MSMS) fragmentation with a collision energy ramp of 10–50 eV was used to assist in determining the structure of metabolites. The performance of LC-MS analysis was monitored by injecting a pooled sample three times (beginning, middle, and end) in each run as quality controls (QC) (Figure S2). The LC-MS data have been deposited to the Metabolomics Workbench databank (https://www.metabolomicsworkbench.org/ (accessed on 1 January 2021)) with their study IDs as ST001657, ST001663, and ST001664.

### 4.10. Multivariate Data Analysis

In total, 497 features were captured by MarkerLynx software (Waters, Milford, MA, USA) and incorporated into a multivariate data matrix after centroiding, deisotoping, filtering, peak recognition, and integration. Sample name, ion feature (retention time and *m*/*z*), and ion abundance were included in the matrix. The relative intensity of each ion was calculated by normalizing the single ion counts (SIC) versus the total ion counts (TIC) in the whole chromatogram, while setting the TIC arbitrarily to 10,000. The processed data matrix was further exported into SIMCA-P^+TM^ software (Umetrics, Kinnelon, NJ, USA), transformed by *Pareto* scaling, and then processed by principal components analysis (PCA). The contribution of samples to the principal components were described in a scores scatter plot of a multivariate model. The IPL-responsive metabolites were identified by analyzing the ions contributing to the principal components in a loadings scatter plot. The chemical identities of metabolite markers were determined by elemental composition analysis, database search (Metlin, http://metlin.scripps.edu/ (accessed on 16 January 2021); ECMDB, http://ecmdb.ca/ (accessed on 16 January 2021); HMDB, www.hmdb.ca (accessed on 16 January 2021); and KEGG, https://www.genome.jp/kegg/ (accessed on 16 January 2021)), fragmentation, and comparison with authentic standards if possible. Elemental composition analysis was conducted using the respective module in MassLynx (Waters, Milford, MA, USA) software, which calculates the molecular formula based on both accurate mass (within 5 ppm of exact mass) and the fitness value based on the similarity between the measured abundances of isotopic peaks and their theoretical abundances.

### 4.11. Statistical Analysis

Statistical analysis was performed by one-way ANOVA and Tukey-Kramer comparison test using the GraphPad Prism 6 (GraphPad Software, La Jolla, CA, USA) for comparing the relative abundances of metabolites across different treatment groups. *P* < 0.05 was considered as statistically significant.

## Figures and Tables

**Table 1:** Detection and identities of IPL-responsive metabolites in the *E. coli* metabolome. The metabolite markers were detected in four different modes, including positive-mode detection of protonated metabolites ([M + H]^+^) in aqueous extracts, negative-mode detection of deprotonated metabolites ([M − H]^−^) in aqueous extracts, positive-model detection of dansylated metabolites ([M + DC]^+^) in aqueous extracts, and positive-mode detection of both protonated ([M + H]^+^) and ammonium ([M + NH_4_]^+^) adducts in lipid extracts.

Ions	Modes of Ion Detection	Detected *m*/*z*	Identity	Formula	Mass Deviation (ppm)	KEGG ID
I	[M − H]^−^	306.0752	Glutathione *	C_10_H_17_N_3_O_6_S	−2.61	C00051
II	[M + H]^+^	118.0867	Valine *	C_5_H_11_NO_2_	−0.85	C00183
II	[M + DC]^+^	351.1369	Valine *	C_5_H_11_NO_2_	−2.85	C00183
III	[M − H]^-^	229.0111	Ribose 5-phosphate *	C_5_H_11_O_8_P	−0.87	C00117
IV	[M − H]^-^	171.0041	Glycerol 3-phosphate *	C_3_H_9_O_6_P	−0.58	C00093
V	[M + H]^+^	704.5238	PE(16:0/17:0Cyclo) ^#,†^	C_38_H_74_NO_8_P	1.14	NA
VI	[M − H]^-^	154.062	Histidine *	C_6_H_9_N_3_O_2_	1.95	C00135
VII	[M + DC]^+^	381.1111	Glutamate *	C_5_H_9_NO_4_	−0.26	C00025
VIII	[M + NH_4_]^+^	746.6103	Ubiquinol-8 ^#^	C_49_H_76_O_4_	2.01	C00390
IX	[M + NH_4_]^+^	734.5888	Menaquinone-8 ^#^	C_51_H_72_O_2_	1.63	C00828
X	[M + H]^+^	727.5684	Ubiquinone-8 ^#^	C_49_H_74_O_4_	2.61	C17569
XI	[M + H]^+^	732.5553	PE(16:0/19:0Cyclo) ^#,†^	C_40_H_78_NO_8_P	1.37	NA
XII	[M + H]^+^	664.4928	PE(14:0/16:0) ^#,†^	C_35_H_70_NO_8_P	1.66	C00350
XIII	[M − H]^−^	364.0802	S-carboxymethyl-glutathione *	C_12_H_19_N_3_O_8_S	−3.57	C14862
XIV	[M + H]^+^	98.9848	Phosphoric acid *	H_3_PO_4_	1.01	C00009
XV	[M − H]^−^	346.0543	Adenosine monophosphate *	C_10_H_14_N_5_O_7_P	−2.89	C00020
XV	[M + H]^+^	348.0701	Adenosine monophosphate *	C_10_H_14_N_5_O_7_P	−2.30	C00020
XVI	[M − H]^-^	323.0269	Uridine monophosphate *	C_9_H_13_N_2_O_9_P	−3.41	C00105
XVII	[M + H]^+^	188.1761	*N*-Acetylspermidine ^#^	C_9_H_21_N_3_O	−1.06	C01029
XVIII	[M + DC]^+^	364.1688	*N*-Acetylputrescine *	C_6_H_14_N_2_O	−1.92	C02714
XIX	[M + DC]^+^	378.1845	*N*-Acetylcadaverine ^#^	C_7_H_16_N_2_O	−1.85	NA

* Confirmed by authentic standard. # Determined by database search and MSMS fragmentation; their major fragments are listed in Table S1. ^†^ PE, phosphatidylethanolamine.

## Data Availability

All data are available in the manuscript and in the Supplementary Material.

## Supplementary Materials

The following are available online at https://www.mdpi.com/2218-1989/11/2/102/s1, Table S1: MSMS fragments of selective metabolite markers, Table S2: Respective mobile phase gradients for 10-min LC runs of *E. coli* extracts, Table S3: MS settings in the ESI detection, Figure S1: Confirmation of S-carboxymethyl-glutathione (CMGSH) in *E. coli* by a comparison with its standard, Figure S2: Scores plot with quality control (QC) samples.
