# Supplementary material for: Identification of Quinone Degradation as a Triggering Event for Intense Pulsed Light-Elicited Metabolic Changes in Escherichia coli by Metabolomic Fingerprinting
Source: Metabolites. 2021 Feb 10;11(2):102. doi: 10.3390/metabo11020102 (PMC7916761; doi:10.3390/metabo11020102)
Supplement: Supplementary file 1 [file metabolites-11-00102-s001.pdf]

Identification of Quinone Degradation as a Triggering Event for Intense Pulsed Light-Elicited Metabolic Changes in *Escherichia coli* by Metabolomic Fingerprinting

Qingqing Mao[1], Juer Liu[1], Justin R. Wiertzema[1], Dongjie Chen[1], Paul Chen[2], David J. Baumler[1], Roger Ruan[2], Chi Chen[1,*]

[1] Department of Food Science and Nutrition, University of Minnesota, 1334 Eckles Ave, Saint Paul, MN 55108, USA; maoxx113@umn.edu (Q.M.); liux3514@umn.com (J.L.); wiert006@umn.edu (J.R.W.); chen5166@umn.edu (D.C.); dbaumler@umn.edu (D.J.B.)

[2] Department of Bioproducts and Biosystems Engineering, University of Minnesota, 1390 Eckles Ave., Saint Paul, MN 55108, USA; chenx088@umn.edu (P.C.); ruanx001@umn.edu (R.R.)

* Correspondence: chichen@umn.edu; Tel.: +1-612-624-7704; Fax: +1-612-625-5272

Supplementary Data

Table S1. MSMS fragments of selective metabolite markers

Ions	Detected Ion Adduct	m/z	Identity	Formula	m/z of major MS/MS fragments
V	[M+H]+	704.5238	PE(16:0/17:0Cyclo)#	$C_{38}H_{74}NO_8P$	563 in ESI$^+$; 255, 267 in ESI$^-$
VIII	[M+NH$_4$]+	746.6103	Ubiquinol-8#	$C_{49}H_{76}O_4$	197
IX	[M+NH$_4$]+	734.5888	Menaquinone-8#	$C_{51}H_{72}O_2$	187
X	[M+H]+	727.5684	Ubiquinone-8#	$C_{49}H_{74}O_4$	197
XI	[M+H]+	732.5553	PE(16:0/19:0Cyclo)#	$C_{40}H_{78}NO_8P$	593 in ESI$^+$; 255, 295 in ESI$^-$
XII	[M+H]+	664.4928	PE(14:0/16:0)#	$C_{35}H_{70}NO_8P$	523 in ESI$^+$; 227, 255 in ESI$^-$
XVII	[M+H]+	188.1761	*N*-Acetylspermidine#	$C_9H_{21}N_3O$	171, 114, 84
XIX	[M+DC]+	378.1845	*N*-Acetylcadaverine#	$C_7H_{16}N_2O$	170, 86

Table S2. Respective mobile phase gradients for 10-min LC runs of *E. coli* extracts

Column	BEH C18	BEH Amide	BEH C8
**Solvent A**	0.1% formic acid in water	0.1% formic acid in water	0.1% formic acid and 10 mM NH_4OAc in 40% aqueous acetonitrile
**Solvent B**	0.1% formic acid in acetonitrile	0.1% formic acid in acetonitrile	0.1% formic acid and 10 mM NH_4OAc in methanol
**Gradient**	0.5 min-99.5% A, 4 min-80% A, 8 min-5% A, 8.1 min-0% A, 9.0 min-0% A, 9.1 min-99.5% A, 10 min-99.5% A	0.5 min-0.5% A, 2 min-20% A, 8 min-50% A, 9.1 min-0.5% A, 10 min-0.5% A	0.5 min-55.5% A, 2.5 min-20% A, 5 min-15% A, 8 min-5% A, 8.1 min-0% A, 9.0 min- 0% A, 9.1 min-55% A, 10 min-55% A

Table S3. MS settings in the ESI detection

**Capillary voltage**	3 kV for ESI$^+$, -3 kV for ESI$^-$
**Cone voltage**	40 V for ESI$^+$, -35 V for ESI$^-$
**Gas flow**	Nitrogen, cone gas 50 L/h, desolvation gas 600 L/h

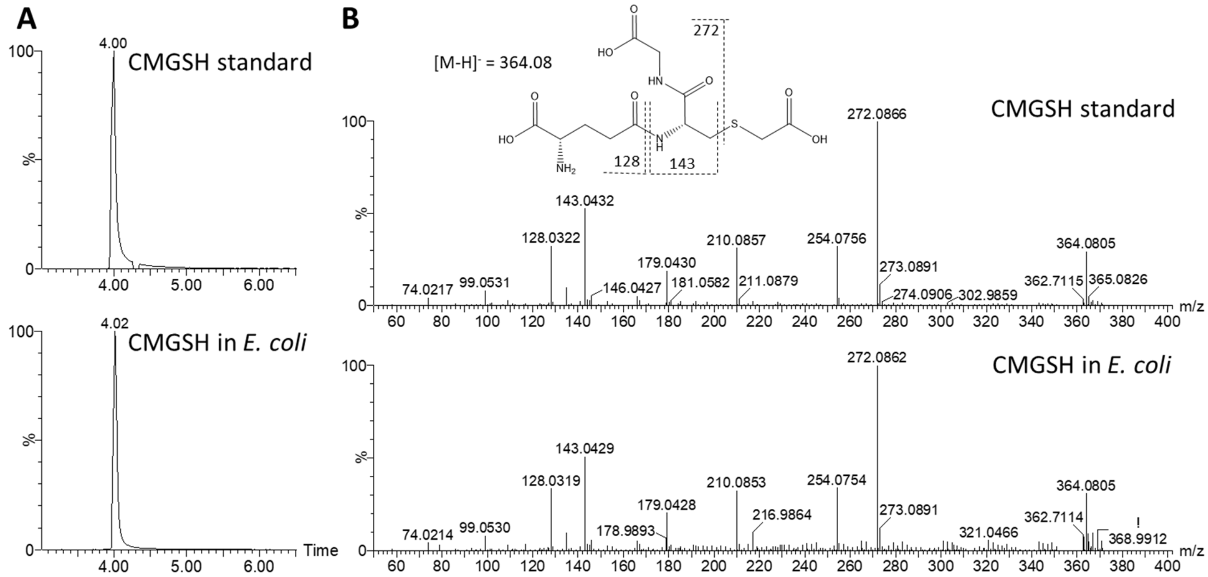

Figure S1. Confirmation of S-carboxymethyl-glutathione (CMGSH) in *E. coli* by a comparison with its standard. The CMGSH standard was synthesized by a reaction between GSH and iodoacetic acid as described in the Materials and Methods. (**A**) Extracted chromatographs of CMGSH standard and CMGSH in the polar extract of IPL-treated *E. coli*. (**B**) MSMS fragmentograms of CMGSH standard and CMGSH in the polar extract of IPL-treated *E. coli*. The fragmentation was conducted at the negative ionization mode and interpreted in the inlaid structure diagram.

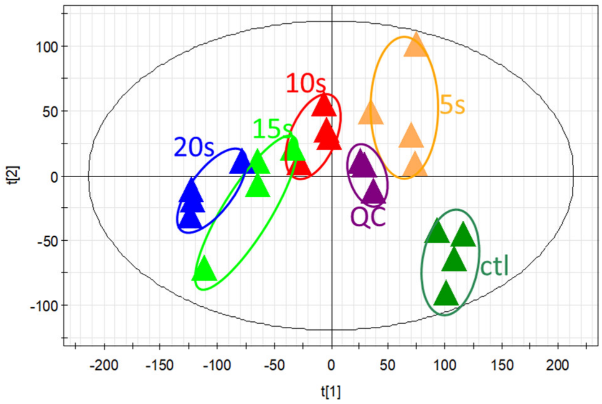

Figure S2. Scores plot with quality control (QC) samples. A pooled sample was injected for a total of three times (beginning, middle, and end) as the QC in each run for monitoring the LC-MS performance.
